# Effect of dietary supplementation of alpha-galactosidase on the growth performance, ileal digestibility, intestinal morphology, and biochemical parameters in broiler chickens

**DOI:** 10.1186/s12917-020-02359-7

**Published:** 2020-05-20

**Authors:** Shimaa A. Amer, Mohamed A. F. Naser, Ahmed A. A. Abdel-Wareth, Ahmed A. Saleh, Shafika A. M. Elsayed, Doaa M. Abdel fattah, Abdallah E. Metwally

**Affiliations:** 1 Department of Nutrition & Clinical Nutrition, Faculty of Veterinary Medicine, Zagazig University, Zagazig, 44511 Egypt; 2 Animal Wealth Development Department, Faculty of Veterinary Medicine, Zagazig University, Zagazig, 44511 Egypt; 3 Department of Animal and Poultry Production, Faculty of Agriculture, South Valley University, Qena, 83523 Egypt; 4 Department of Poultry Production, Faculty of Agriculture, Kafr Elsheikh University, Kafr Elsheikh, 33516 Egypt; 5 Department of Histology and Cytology, Faculty of Veterinary Medicine, Zagazig University, Zagazig, 44511 Egypt; 6 Department of Biochemistry, Faculty of Veterinary Medicine, Zagazig University, Zagazig, 44511 Egypt

**Keywords:** Broiler chicken, Alpha-Galactosidase, Growth performance, Ileal digestibility, Gut health, Economic value

## Abstract

**Background:**

This study was performed to investigate the effect of Alpha-galactosidase (AlphaGal) supplementation with two energy levels on the growth performance, amino acid ileal digestibility coefficient “AID%,” economic value, intestinal histology, and blood biochemical parameters of broiler chickens. Two-hundred 3-day-old broiler chicks (average body weight 74.34 g ±0.52 Ross 308) were randomly assigned to a 2 × 2 factorial arrangement consisting of two energy diets groups: in the first group, the birds were fed on a recommended energy diet (RED) while the second group was reduced 120 kcal/kg diet as a low energy diet (LED) and two levels of AlphaGal (0 or 50 mg/kg diet) for RED and LED for the 35-day feeding period.

**Results:**

The interaction effects between the energy level and the AlphaGal supplementations resulted in significant decrease (*P* ≤ 0.05) in the body weight, body weight gain, and the relative growth rate. The feed conversion ratio was signficantly increased in LED without supplementation of AlphaGal group during the entire experimental period, this negative effect on the growth performance was corrected by AlphaGal supplementation. The AID% value was increased significantly by AlphaGal supplementation. Blood triglyceride concentrations were significantly decreased (*P* = 0.02) in the LED group with or without AlphaGal supplementation, while the level of high-density lipoprotein (HDL) was significantly decreased (*P* = 0.01) in the LED or RED groups supplemented with 50 mg RED AlphaGal. Histologically, the number of intestinal glands and goblet cells increased in both RED and LED groups supplemented with AlphaGal and their secretions were mainly neutral mucopolysaccharides and less acidic mucopolysaccharides.

**Conclusion:**

AlphaGal supplementation improved the growth performance of broiler chickens fed LED and the growth performance is similar to those fed RED, thereby consequently improving the economic value of these diets. AlphaGal supplementation improves intestinal histology and morphology as well.

## Background

Poultry diets are prepared using corn and soybean meal “SBM” as the major source of energy and protein. Although SBM has higher gross energy than corn, its metabolizable energy is less than that of corn [1]. This is due to the higher content of α-galactosides (raffinose and stachyose) in SBM that is indigestible by poultry due to lack of digestive enzymes exhibiting α-galactosidase (AlphaGal) activity [2]. α-galactosides are heat resistant and are considered anti-nutritional factors [3, 4]. Feeding chicks with such compounds in SBM-based diets resulted in a reduction in energy utilization, fiber digestion, and nutrient retention [3]. Dietary supplementation of an exogenous enzyme preparation, composed mainly of AlphaGal, can reduce the negative effects of α-galactosides [5, 6]. By supplementing corn-SBM diets with AlphaGal, the α-(1 → 6)-glycosidic linkages will be broken giving sucrose and galactose, which may be utilized for providing partial energy and consequently, eliminating their negative effects [7, 8].

Several studies have demonstrated that feeding the broiler chicken corn-SBM based diets supplemented with AlphaGal resulted in significant improvement in energy utilization and nutrient bioavailability that sequentially resulted in improved growth performance and reduced intestinal viscosity [9–14] as well as the energy value of feeds and nutrient bioavailability [15–18]. Ethanol extraction of oligosaccharides has been reported to result in significant improvement in the metabolizable energy content of SBM fed to adult roosters [19]. G Marsman, H Gruppen, A Van der Poel, R Kwakkel, M Verstegen and A Voragen [20]. There are possible reasons for the variable responses to dietary supplementation of AlphaGal in poultry and swine include enzyme source, enzyme specificity on the key substrate (stachyose and raffinose), supplementary actions of the products, SBM source, optimal pH, and environmental conditions [21].

Therefore, this study aims to evaluate the effect of AlphaGal supplementation to two diets with different energy levels and their effect on growth performance, apparent ileal digestibility (AID) coefficient of amino acids, economic value, intestinal histology, and a few vital blood biochemical parameters.

## Results

### Growth performance

As shown in Table 1, there were no significant differences (*P* > 0.05) in the growth performance parameters between the RED and LED groups for the entire experimental period. Body weight (BW) and body weight gain (BWG) during the grower period (11 to 23 days) and body weight only during the finisher period (24 to 35 days) increased significantly in the AlphaGal-supplemented group compared to the non-supplemented group (*P* < 0.05). The final body weight, total body weight gain, protein efficiency ratio (PER), and relative growth rate (RGR) significantly increased (P < 0.05) and the overall feed conversion ratio (FCR) were decreased significantly by AlphaGal supplementation. The results also indicated an association between the feed energy level and the presence of AlphaGal where insignificant decrease in BW, BWG, and RGR was observed during the entire experimental period (*P* < 0.05) and a significant increase was observed (P < 0.05) in the overall FCR in the LED+ 0AlphaGal group compared to RED+ 0AlphaGal, RED+ 50AlphaGal, and LED+ 50AlphaGal groups. AlphaGal supplementation, therefore, corrects the negative effect of a low energy diet on the growth performance of broiler chickens. There was no significant difference in BW, BWG, FCR, PER, and RGR values between the LED+ 50AlphaGal and RED+ 0AlphaGal groups. No significant difference was found in PER and FI values during the entire experimental period among all experimental groups.

**Table 1:** The effect of dietary supplementation of AlphaGal to normal and low energy diet on the growth performance parameters of broiler chickens

Item	Energy		*P*-value	AlphaGal (mg/kg diet)		*P*-value	Energy × AlphaGal				*P-value*	SEM
	RED	LED		0	50		RED+ 0 AlphaGal	RED+ 50 AlphaGal	LED+ 0 AlphaGal	LED+ 50 AlphaGal		
Initial wt (gm)	74.58	74.00	0.63	74.41	74.16	0.83	74.50	74.66	74.33	73.66	0.95	0.57
Starter period												
BW (gm)	209.38	203.16	0.39	199.22^b^	213.33^a^	0.03	203.44	215.33	195.00	211.33	0.15	3.43
BWG (gm)	134.80	129.16	0.43	124.80^b^	139.16^a^	0.02	128.94	140.66	120.66	137.66	0.12	3.37
FI (gm)	188.22	192.66	0.7	188.05	192.83	0.68	181.77	194.66	194.33	191.00	0.86	5.41
FCR	1.39	1.49	0.17	1.50	1.38	0.066	1.41^b^	1.38^b^	1.60^a^	1.38^b^	0.03	0.03
Grower period												
BW (gm)	910.44	871.83	0.26	851.77^b^	930.50^a^	0.009	887.22^a^	933.66^a^	816.33^b^	927.33^a^	0.01	16.62
BWG (gm)	701.05	668.66	0.28	652.55^b^	717.16^a^	0.016	683.77^ab^	718.33^a^	621.33^b^	716.00^a^	0.025	14.43
FI (gm)	966.77	970.00	0.93	939.94	996.83	0.10	947.88	985.66	932.00	1008.00	0.44	17.47
FCR	1.38	1.45	0.16	1.44	1.39	0.3	1.38	1.37	1.50	1.40	0.31	0.025
Finisher period												
BW (gm)	2032.25	1924.50	0.185	1900.58^b^	2056.16^a^	0.04	2006.16^a^	2058.33^a^	1795.00^b^	2054.00^a^	0.023	39.53
BWG (gm)	1121.80	1052.66	0.23	1048.80	1125.66	0.18	1118.94	1124.66	978.66	1126.66	0.16	28.16
FI (gm)	1996.88	1917.66	0.19	1966.22	1948.33	0.77	2043.44	1950.33	1889.00	1946.33	0.35	29.49
FCR	1.79	1.82	0.68	1.88	1.73	0.1	1.83	1.74	1.93	1.72	0.37	0.043
Overall performance												
BW (gm)	2032.25	1924.50	0.185	1900.58^b^	2056.16^a^	0.04	2006.16^a^	2058.33^a^	1795.00^b^	2054.00^a^	0.023	39.53
BWG (gm)	1957.66	1850.50	0.18	1826.16^b^	1982.00^a^	0.04	1931.66^a^	1983.66^a^	1720.66^b^	1980.33^a^	0.02	39.46
FI (gm)	3151.88	3080.33	0.42	3094.22	3138.00	0.63	3173.11	3130.66	3015.33	3145.33	0.63	42.59
FCR	1.61	1.66	0.29	1.69^a^	1.58^b^	0.023	1.64^ab^	1.58^b^	1.75^a^	1.58^b^	0.04	0.026
PER	2.99	2.88	0.25	2.83^b^	3.03^a^	0.03	2.93	3.04	2.74	3.02	0.06	0.04
RGR	185.80	185.12	0.29	184.86^b^	186.06^a^	0.02	185.36^a^	185.97^a^	184.09^b^	186.14^a^	0.01	0.29

^a,b,c,d)^Means within the same row carrying different superscripts are significantly different at (*P < 0.05*). *RED* Recommended energy diet, *LED* Low energy diet “–120 kcal/kg diet”

*BW* Body weight, *BWG* Body weight gain, *FI* Feed intake, *FCR* Feed conversion ratio, *PER* Protein efficiency ratio, *RGR* Relative growth rate

### Determining the apparent ileal digestibility coefficient of amino acids

The effect of dietary supplementation of AlphaGal on the AID coefficient (%) of amino acids is shown in Table 2. Generally, the energy level of the diets had no significant effect on the ileal digestibility coefficient of amino acids except for the AID% of leucine that had the highest percentage (*P* < 0.05) found in RED. AlphaGal supplementation significantly increased the AID% of lysine, methionine, threonine, tryptophan, arginine, valine, and isoleucine (*P* < 0.05). AlphaGal supplementation resulted in a significant increase (*P* < 0.05) in the AID% of lysine, methionine, threonine, arginine, valine in AlphaGal-supplemented diets and a significant increase (*P* < 0.05) in the AID% of arginine and isoleucine only in the LED+ 50AlphaGal group. The AID% of threonine decreased significantly (*P* = 0.03) in the LED+ 0AlphaGal group.

**Table 2:** The effect of dietary supplementation of AlphaGal to normal and low energy diet on the apparent ileal digestibility coefficient (AID%) of amino acids

Item	Energy		*P*-value	AlphaGal (mg/kg diet)		*P*-value	Energy × AlphaGal				*P*-value	SEM
	RED	LED		0	50		RED+ 0 AlphaGal	RED+ 50 AlphaGal	LED+ 0 AlphaGal	LED+ 50 AlphaGal		
Lysine	89.06	89.19	0.49	86.76^b^	87.34^a^	0.00	86.79^b^	87.46^a^	86.73^b^	87.22^a^	0.002	0.09
Methionine	87.12	86.98	0.43	88.89^b^	89.35^a^	0.00	88.89^c^	89.23^b^	88.89^c^	89.48^a^	0.00	0.07
Threonine	84.89	84.72	0.40	84.52^b^	85.09^a^	0.00	84.70^b^	85.09^a^	84.34^c^	85.10^a^	0.00	0.09
Tryptophan	87.27	87.08	0.60	86.82^b^	87.53^a^	0.02	87.03	87.51	86.62	87.55	0.12	0.16
Arginine	90.03	90.12	0.28	89.98^b^	90.17^a^	0.004	89.94^b^	90.13^ab^	90.02^ab^	90.21^a^	0.01	0.03
Valine	85.00	85.13	0.28	84.90^b^	85.23^a^	0.001	84.83^c^	85.16^ab^	84.97^bc^	85.30^a^	0.004	0.05
Leucine	91.06^a^	90.33^b^	0.00	90.75	90.64	0.65	91.11^a^	91.01^a^	90.38^b^	90.27^b^	0.00	0.11
Isoleucine	86.17	86.25	0.42	86.09^b^	86.33^a^	0.01	86.11^b^	86.23^ab^	86.07^b^	86.43^a^	0.02	0.05

^a,b,c,d)^Means within the same row carrying different superscripts are significantly different at (*P < 0.05*)

*RED* Recommended energy diet, *LED* Low energy diet “–120 kcal/kg diet”

### Blood biochemical parameters

The effect of dietary supplementation of AlphaGal on lipid profile is shown in Table 3.There was a significant decrease (P < 0.05) in the blood of TG and HDL levels in the LED groups compared with the RED groups. AlphaGal supplementation decreased significantly the blood level of HDL (*P* = 0.001). The effect of an association between the energy level and presence of AlphaGal led to a significant decrease in the blood level of TG (*P* = 0.02) in the LED+ 0AlphaGal and LED+ 50AlphaGal groups, while the HDL level was significantly decreased (*P* = 0.01) in the LED+ 0AlphaGal, LED+ 50AlphaGal, and RED+ 50AlphaGal groups. No significant differences were observed in the levels of total cholesterol and LDL between the RED and other groups.

**Table 3:** The effect of dietary supplementation of AlphaGal to low energy diet on the immune status and lipid profile of broiler chickens

Item	Energy		*P*-value	AlphaGal (mg/kg diet)		*P*-value	Energy × AlphaGal				*P*-value	SEM
	RED	LED		0	50		RED+ 0 AlphaGal	RED+ 50 AlphaGal	LED+ 0 AlphaGal	LED+ 50 AlphaGal		
Alkaline phosphatase (U/L)	55.21^b^	84.950^a^	0.02	70.51	69.65	0.93	57.49	60.27	90.86	79.03	0.34	6.14
Immune response												
IgM (mg/dL)	99.84	89.55	0.796	114.23	75.16	0.31	94.77	103.00	131.76	47.33	0.45	18.53
Complement 3 (mg/dL)	89.67	102.69	0.514	110.12	82.24	0.14	110.00	78.01	118.91	86.48	0.405	9.84
Interleukin 10 (pg/mL)	0.18	0.15	0.44	0.16	0.17	0.69	0.14	0.22	0.17	0.13	0.43	0.02
Lipid profile												
TG (mg/dL)	94.74^a^	61.32^b^	0.01	86.58	69.47	0.14	122.45^a^	75.97^ab^	59.66^b^	62.98^b^	0.02	9.63
TC (mg/dL)	202.86	218.64	0.55	215.69	205.81	0.68	204.29	194.96	220.61	216.66	0.82	10.88
LDL (mg/dL)	127.31	131.85	0.813	129.75	129.41	0.98	133.63	124.22	129.09	134.61	0.979	10.10
HDL (mg/dL)	60.58^a^	50.77^b^	0.02	64.98^a^	46.37^b^	0.001	70.93^a^	45.88^b^	54.67^b^	46.87^b^	0.01	2.06

^a,b,c,d)^Means within the same column carrying different superscripts are significantly different at (*P < 0.05*)

*RED* Recommended energy diet, *LED* Low energy diet “–120 kcal/kg diet”

*TC* Total cholesterol, *TG* Triglycerides, *HDL* High-density lipoprotein, *LDL* Low-density lipoprotein

Table 3 also highlights the effect of dietary supplementation of AlphaGal on blood IgM, interleukin 10, complement 3, and alkaline phosphatase levels. The results revealed a significant increase (*P* = 0.02) in the level of alkaline phosphatase in the LED groups compared with the RED groups. AlphaGal supplementation had no significant effect on the blood levels of IgM, complement 3, interleukin 10, and alkaline phosphatase in all the experimental groups.

### Histological observations

**Fig. 1:**
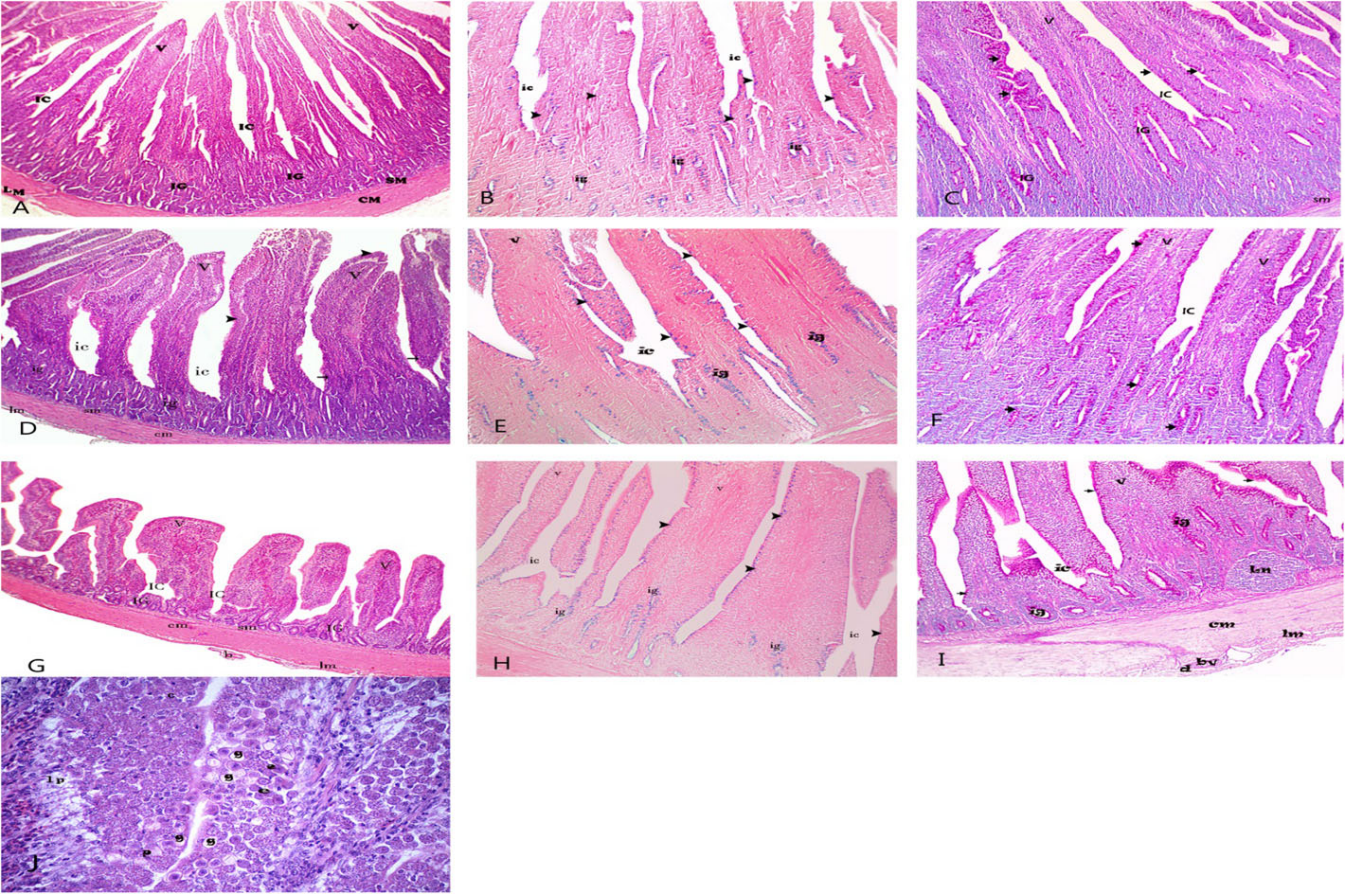
Showed intestinal sections from RED+0AlphaGal group. **a** (duodenum), **d** (jejunum), **g** (ileum): showed long villi in both duodenum & jejunum while short one in ileum(v), intestinal crypt (ic), chief columnar cell (arrow head), goblet cell (arrow) intestinal glands (iG), submucosa (sm) tunica muscularis circular layer (cm), longitudinal layer(lm) blood vessel(b) H&E X40. **j** showed goblet cell (g), panth cell (p), endocrine cell (e) and lamina propria (lp) H&E X400. **b** (duodenum), **e** (jejunum), **h** (ileum) showed weak reaction for Alcian blue in goblet cell (arrow head) & intestinal glands (ig) X100. **c** (duodenum), **f** (jejunum), **i** (ileum) showed positive PAS reaction in goblet cell (arrow head) & intestinal glands (ig) X100

Sections from the small intestine revealed a normal intestinal wall in the RED+ 0AlphaGal group. The height of the villi of both duodenum and jejunum samples was the longest in the RED+ 0AlphaGal group while the villi became shorter and broader in the ileum. Each villus was covered by simple columnar chief cells with abundant goblet cells (Fig. 1a, d, g). A strong magenta coloration of the goblet cells to PAS was observed in the mucosa of all small intestine segments (Fig. 1c, f, i) while Alcian blue staining produced weak bluish coloration (Fig. 1b, e, h). These reactions indicated that the cells mainly secreted neutral mucopolysaccharides.

**Fig. 2:**
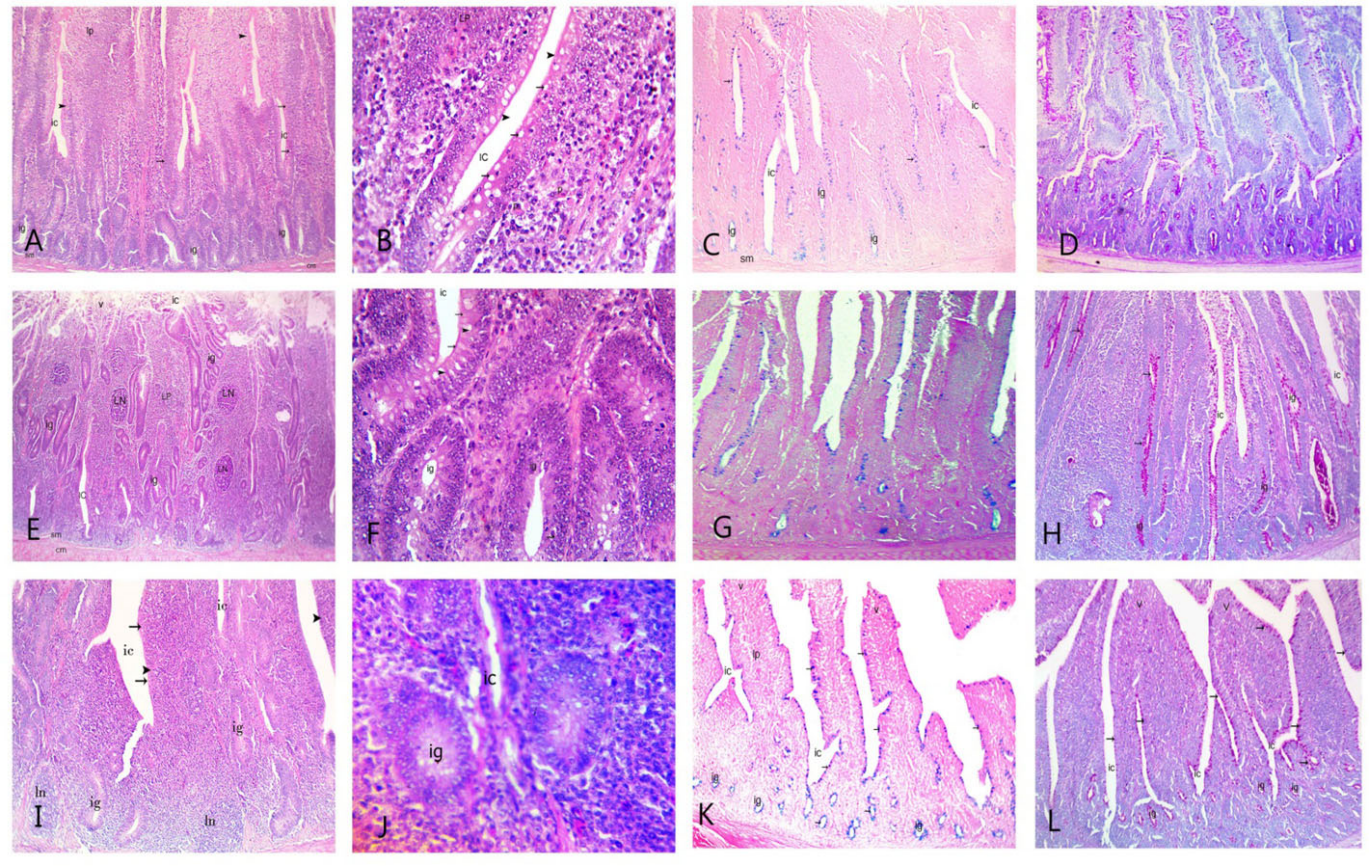
Showed intestinal sections from LED+ 0AlphaGal group. **a** (duodenum), **e** (jejunum), **i** (ileum): showed intestinal crypt (ic) lined with columnar cells (arrow head), goblet cell (arrow), increasing intestinal glands (ig),also lymph nodules (ln) in lamina propria (lp) in **e** “H&E X40”. **b**, **f**, **j** mag. of A, E, I X400. **c**, **g**, **k** showing intestinal crypt (ic),weak reaction for Alcian blue in goblet cell (arrow), intestinal glands (ig) in **c** (duodenum),and more in **g** (jejunum), and **k** (ileum) Alc. bl. X 100. **d**, **h**, **l** showed intestinal crypt (ic),moderate reaction for PAS in in goblet cell (arrow), intestinal glands (ig) in **d** (duodenum), **l** (ileum) and strong in **h** (jejunum) PAS X100

The jejunum of the LED+ 0AlphaGal group showed an increase in the number of intestinal glands, in addition to many lymph nodules in lamina propria. There was a slight increase in goblet cell numbers. The ileum showed the highest length of the villi and maximum crypt depth (Fig. 2a, e, i, b, f, g). The villi and glands of the duodenum samples showed weak staining after treatment with Alcian blue, while the jejunum and ileum were strongly stained (Fig. 2c, g, k), indicating that there was a strong reaction for PAS in the duodenum and ileum but it was the strongest in the jejunum (Fig. 2d, h, l).

**Fig. 3:**
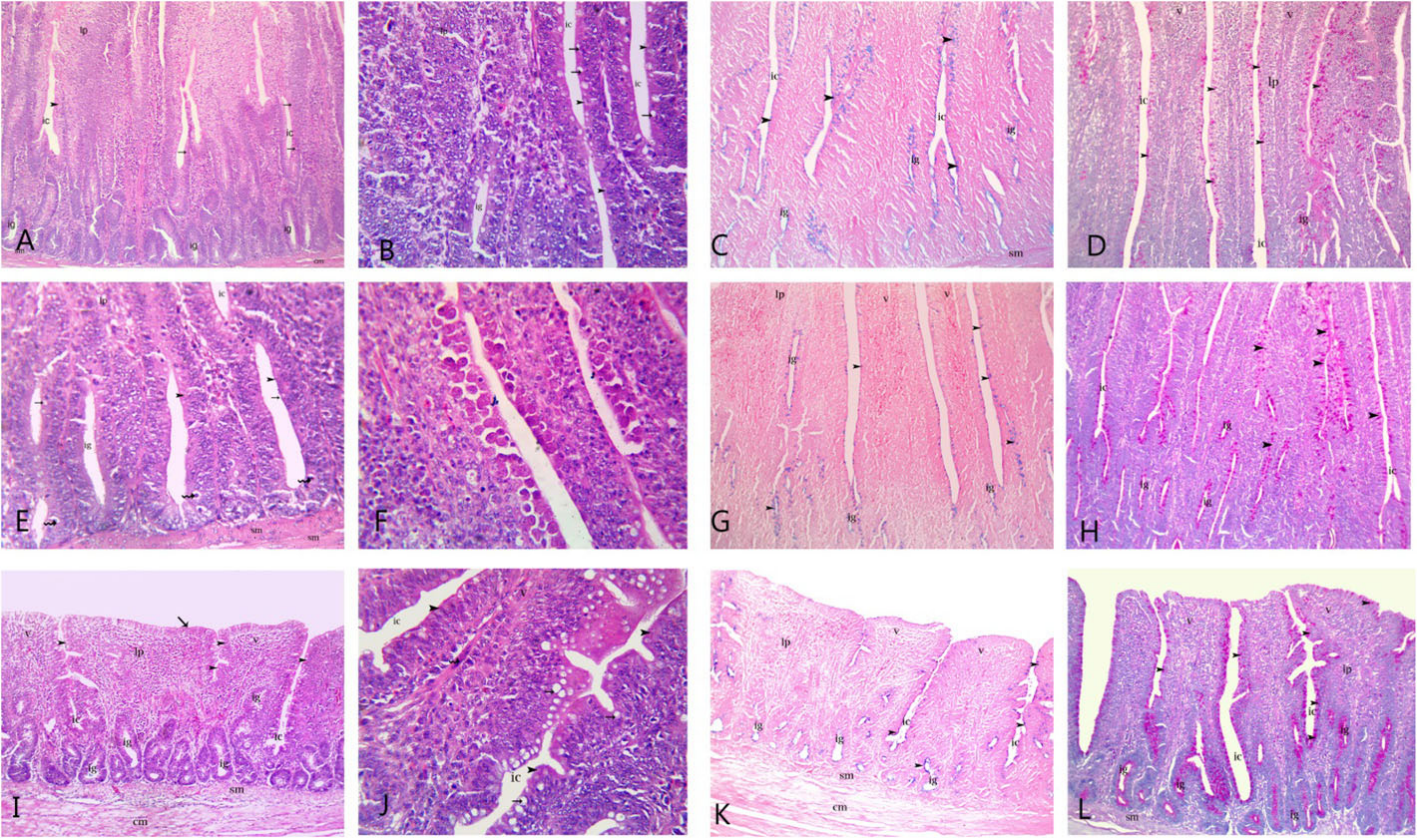
Showed intestinal sections from RED+50AlphaGal group. **a** (duodenum), **e** (jejunum), **i** (ileum): showing intestinal crypt (ic) lined with columnar cells (arrow head),&goblet cell (arrow) and endocrine cell (zigzag arrow) increasing intestinal glands (ig) **b**, **f**, **j** mag. of **a**, **e**, **i** (H&E X400). **c** (duodenum), **g** (jejunum) and **k** (ileum) showed weak reaction for Alcian blue in goblet cell (arrow head), intestinal glands (ig). Alcian blue C&G X100, K X40. **d**, **h, l** showed strong PAS reaction in goblet cell (arrow head) and intestinal glands (ig). **d** (duodenum), **h** (jejunum), and more strong in **l** (ileum) PAS X100

Regarding the RED+ 50AlphaGal group, there was a slight improvement in intestinal tissue architecture. There was an increase in the crypt depth of the duodenum in this group. The villi of the ileum were found to be broader and there was an increase in the number of goblet cells. The reactions for Alcian blue and PAS for the RED+ 50AlphaGal group were shown in (Fig. 3a–l).

**Fig. 4:**
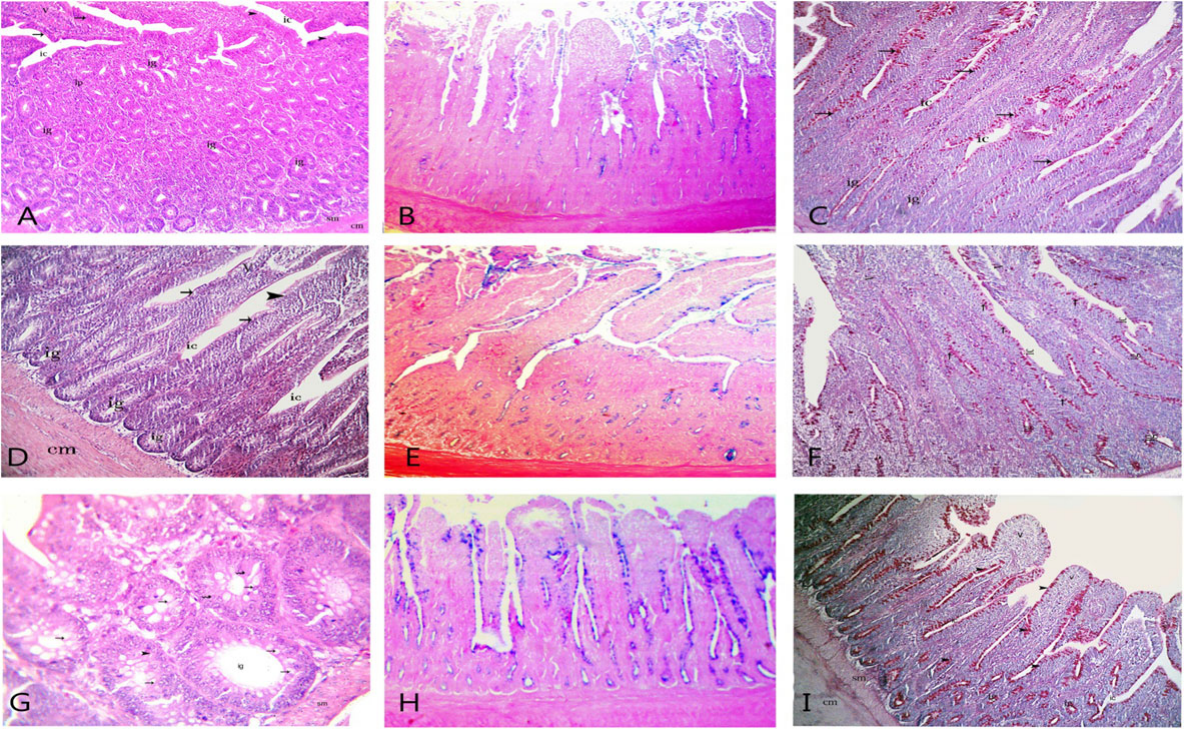
Showed intestinal sections from LED+ 50AlphaGal group. **a** (duodenum), **d** (jejunum), **g** (ileum): showed intestinal villi (v) lined by columnar cell (arrow head) and goblet cells (arrow). Also intestinal crypts (ic) lined with the same cells and endocrine cell (zigzag arrow), circular muscle (cm). **a** and **d** “H&E X40” and X100 for G. **b** (duodenum), **e** (jejunum) presented moderate reaction for Alcian blue while, **h** (ileum) is stronger than B&E Alcian blue X 100. **d**, **h**, **l** showed strong PAS reaction in goblet cell (arrow) and intestinal glands (ig) PAS tech. X100

Sections from the LED+ 50AlphaGal group revealed a significant increase in the number of intestinal glands and goblet cells in the intestinal wall mucosa (Fig. 4a, d, g). The reaction for PAS and Alcian blue was more positive in this group than in the RED+ 0AlphaGal group (Fig. 4b, c, e, f, h, i).

### Morphometric measures of the small intestine

The effect of the energy level of the diets and AlphaGal supplementation and their interaction on the morph-metric measures of the small intestine of broiler chickens is summarized in Table 4. The results showed a significant increase (*P* = 0.00) in the duodenal and jejunal villus length and crypt depth in the RED and AlphaGal-supplemented groups. The villous length and crypt depth of the ileum were found to increase (*P* = 0.00) in AlphaGal-supplemented diets. There was no significant effect of the energy level on the villous length and crypt depth of the ileum.

**Table 4:** The effect of dietary supplementation of AlphaGal to normal and low energy diets on the morph-metric measures of the small intestine of broiler chickens

Item	Energy		*P*-value	AlphaGal (mg/kg diet)		*P*-value	Energy × AlphaGal				*P*-value	SEM
	RED	LED		0	50		RED+ 0 AlphaGal	RED+ 50 AlphaGal	LED+0 AlphaGal	LED+50 AlphaGal		
Duodenum												
Villous tall	1527.50^a^	1376.00^b^	0.00	1358.66^b^	1544.83^a^	0.00	1510.66^a^	1544.33^a^	1206.66^b^	1545.33^a^	0.00	43.40
Crypt depth	487.00^a^	326.10^b^	0.02	338.16^b^	474.93^a^	0.00	480.00^a^	494.00^a^	196.33^b^	455.86^a^	0.00	37.04
Jejunum												
Villous tall	1278.66^a^	1052.33^b^	0.03	1069.50^b^	1261.50^a^	0.00	1287.66^a^	1269.66^a^	851.33^b^	1253.33^a^	0.00	56.14
Crypt depth	532.50^a^	355.50^b^	0.05	351.16^b^	536.83^a^	0.00	526.00^a^	539.00^a^	176.33^b^	534.66^a^	0.00	46.90
Ileum												
Villous tall	1437.16	1416.33	0.91	1140.50^b^	1713.00^a^	0.00	1099.00^b^	1775.33^a^	1182.00^b^	1650.66^a^	0.00	90.28
Crypt depth	191.66	199.83	0.93	160.50^b^	231.00^a^	0.00	155.66^b^	211.00^a^	165.33^b^	234.33^a^	0.00	0.11

^a,b)^Means within the same column carrying different superscripts are significantly different at (*P < 0.05*)

*RED* Recommended energy diet, *LED* Low energy diet “–120 kcal/kg diet”

### Economic efficiency of the diets

As shown in Table 5, dietary AlphaGal supplementation resulted in a significant increase (*P* < 0.05) in the total return, net profit, economic efficiency, and performance index% and a significant decrease (*P* = 0.02) in the feed cost/kg gain compared with non-supplemented groups. Generally, there was no significant effect of the energy level on the economic efficiency of the diets. Association between the energy level of the diets and AlphaGal supplementation revealed a significant decrease (P < 0.05) in the total return, net profit, and performance index in the LED+ 0AlphaGalgroup compared to RED+ 0AlphaGal, RED+ 50AlphaGal, and LED+ 50AlphaGalgroups.

**Table 5:** The effect of dietary supplementation of AlphaGal to low energy diet on the economic efficiency of broiler chicken diets

Item	Energy		*P*-value	AlphaGal (mg/kg diet)		*P*-value	Energy × AlphaGal				*P*-value	SEM
	RED	LED		0	50		RED+0 AlphaGal	RED+50 AlphaGal	LED+0 AlphaGal	LED+50 AlphaGal		
Total return (LE)/bird	45.03	42.56	0.18	42.00^b^	45.59^a^	0.04	44.42^a^	45.62^a^	39.58^b^	45.55^a^	0.02	0.908
Net profit (LE)	19.27	17.25	0.2	16.60^b^	19.92^a^	0.02	18.55^a^	19.99^a^	14.64^b^	19.86^a^	0.01	0.770
Total costs (LE)	25.75	25.31	0.39	25.40	25.66	0.62	25.87	25.64	24.93	25.69	0.62	0.248
Feed costs (LE)	18.25	17.81	0.39	17.90	18.16	0.62	18.37	18.19	17.43	18.14	0.62	0.248
Economic efficiency	1.06	0.97	0.27	0.93^b^	1.10^a^	0.01	1.01^ab^	1.10^a^	0.84^b^	1.09^a^	0.03	0.039
Feed cost/kg gain (LE)	9.34	9.66	0.31	9.82^a^	9.17^b^	0.02	9.51^ab^	9.16^b^	10.13^a^	9.18^b^	0.048	0.151
Performance index%	121.88	111.48	0.2	107.97^b^	125.40^a^	0.02	117.68^a^	126.08^a^	98.25^b^	124.72^a^	0.01	4.004

^a,b,c,d)^Means within the same row carrying different superscripts are significantly different at (*P < 0.05*)

*RED* Recommended energy diet, *LED* Low energy diet “–120 kcal/kg diet”

## Discussion

Supplementation of AlphaGal in monogastric feeds where SBM is used as the main protein source has hypothetical potential. AlphaGal preparations have been effectively used in corn-based SBM diets in birds [22, 23]. The results of the present study indicated a positive effect of AlphaGal supplementation on the final body weight, total body weight gain, protein efficiency ratio, relative growth rate, and overall feed conversion ratio. The results also showed the presence of an interaction between the energy level and presence of AlphaGal where its supplementation corrects the negative effect of low energy diet on the growth performance of broiler chickens. These results may be attributed to the role of AlphaGal supplementation in improving the ileal digestibility percentage of amino acids as shown in our results. In addition to improving intestinal histology and gut health, AlphaGal supplementation also improved growth performance and consequently increased the total return, net profit, economic efficiency, and performance index of the feeds and caused a significant decrease in the feed cost/kg gain compared with non-supplemented groups. AlphaGal supplementation corrects the negative effect of a low energy diet on the economic value of the diet.

From a nutritional point of view, AlphaGal supplementation can improve the economic efficiency of poultry diets as a result of the spare effect of liberated protein or amino acids induced by GAL on supplemented levels of proteins and crystalline amino acid [24]. Wang et al. [24] reported a significant increase in the TMEn, TAAA of methionine and cystine, and apparent digestibility of most of the nutrients in GAL-supplemented groups especially in the early stage of growth and resulted in an improvement in growth performance. They also reported no association between the energy level of the diet and GAL supplementation. A significant improvement in energy utilization, FCR, and weight gain of broilers fed soybean meal-based diets supplemented with AlphaGal were reported in [I Knap, A Ohmann and N Dale [22, 23, 25]. The study of [Ghazi et al. [26]] indicated increased nitrogen retention and TME of SBM after GAL supplementation. In contrast, Irish et al. [27] reported no significant effect of GAL on the nutritive value of SBM for broilers under normal temperature. Moreover, Igbasan et al. [28] reported no significant influence of GAL supplementation on growth. The results of Ao et al. [29] indicated a significant increase in body weight gain, feed intake, and increased crude protein level, AMEn, and crude fiber retention and reduced sugar content of the crop by dietary AlphaGal supplementation. The results of Swift et al. [30] indicated that feeding broiler chickens on a diet supplemented with enzyme preparation containing protease, amylase, and α-galactosidase of fungal origin resulted in an improvement in growth performance, and nitrogen and energy digestibility. Zanella et al. [13] reported an improvement in body weight gain and FCR in broilers fed on corn-SBM-based diet supplemented with exogenous carbohydrase preparations because of improved ileal digestibility of proteins and non-starch polysaccharides. Although Marsman et al. [20] found that supplementing SBM-based diets of broilers with protease and carbohydrase only results in improved apparent ileal digestibility of crude proteins and non-starch polysaccharides but did not improve growth performance. Similarly, the results of [Kocher et al. [31], Graham et al. [32, 33] indicated that SBM treatment with AlphaGal improved the digestibility of raffinose, stachyose, and energy without significant improvement in chick growth performance. Graham, et al. [32] showed that treating SBM with AlphaGal increased its TME by 11.9%. Bedford [34] attributed the improvement in ileal protein digestibility to partial depolymerization. The disruption of the cell wall matrix led to the release of entrapped protein and easy access of endogenous proteolytic enzymes. Schang [35] observed an improvement in chick growth performance by AlphaGal supplementation to a low-nutrient-density diet but not to a high-nutrient-density diet.

In contrast, Ao et al. [29] showed that AlphaGal supplementation can improve growth performance by improving the apparent metabolizable energy “AMEn” of the diets, and its addition to the low energy diet increased the AMEn by about 3%. AMEn improvement was attributed to the increase in the digestibility of NSP in the jejunum and the ileum and the effects of the enzymes depending on their type and their inclusion [23, 31]. In the study of Knap et al. [22], dietary supplementation of AlphaGal to the SBM-based diet of adult Leghorn roosters and Arbor Acres broilers was evaluated wherein they reported an improvement in the TMEn of Leghorns and feed conversion of broilers. On the other hand, Irish et al. [27] reported no improvement in the nutrient composition of SBM after removal of 90% of the α-galactosides of sucrose in white Leghorn roosters and broiler chicks fed on corn-based SBM diets. Kidd et al. [18] reported no improvement in the body weight or FCR of chicks fed on a diet supplemented with AlphaGal until 28 days of age. Knap et al. [22] reported improvement in chick performance when AlphaGal was added to corn-based SMB diets and diets contained lupin “alternating vegetable protein sources” in Arbor Acres broilers.

Regarding the effect of feed energy or AlphaGal supplementation or their interaction on the blood biochemical parameters, limited studies have been reported so far. Our results showed a decrease in triglyceride levels in the LED group and decreased HDL levels in AlphaGal-supplemented groups. The alkaline phosphatase level was increased in the LED group. The association between feed energy and AlphaGal caused a non-significant effect on alkaline phosphatase levels. The study of Wang et al. [24] reported increased triglyceride concentration on the 21st day in the high energy group and increased total cholesterol level by AlphaGal addition on the 35th day. The results of the present study showed that neither energy level nor AlphaGal supplementation nor their interaction had a significant effect on the immune response of broiler chickens. Kidd et al. [23] reported that feeding broiler chickens with diets supplemented with an enzyme preparation mainly containing AlphaGal did not have a significant effect on the immunity and longevity of broilers both under normal or warm temperature conditions. However, their past research, Kidd. et al. [18] showed an improvement in the immune response of broilers by AlphaGal supplementation under high-temperature conditions. They attributed these variables results to the difference in the environmental temperature between the two experiments.

In the present study, the intestinal glands were well developed and there was an increase in the number of goblet cells and their secretions in AlphaGal-supplemented groups; these results are comparable with those of [36], who reported that nutrient digestibility in broiler chicken was improved as a result of an increase in the number of peptides, amino acids, and glucose transporters and also in enzyme production.

## Conclusions

From the obtained results, it could be concluded that dietary supplementation of AlphaGal improves the growth performance and ileal digestibility of amino acids in broiler chickens fed on a low energy diet wherein these chickens showed the same growth performance as those fed on a normal energy diet, thereby improving the economic value of the diets. AlphaGal supplementation does not improve the immune response of broiler chickens but it does improve their intestinal histology and morphology.

## Methods

### Birds

Two hundred three-day-old chicks (Ross 308 broiler) were procured from a commercial chick producer (Dakahlia Poultry, Mansora, Egypt) and were used in the experiments. Chicks were subjected to a 3-day adjustment period before the start of the experiment and attained an average body weight of 74.34 g ± 0.52. This experiment was performed in the poultry research unit in the faculty of veterinary medicine, Zagazig University, Egypt. The experimental protocol was approved by the Ethics of the Institutional Animal Care and Use Committee of Zagazig University, Egypt (ZUIACUC–2019) and all animal experiments were performed in accordance with recommendations described in “The Guide for the Care and Use of Laboratory Animals in scientific investigations”. The chicks were reared at 10 bird/m^2^ stocking density. The light regimen in all the experimental pens was maintained at 23 L: 1 D h for the first 3 days, and then 20 L:4 D until the end of the experiment. The initial ambient temperature was about 32 °C during the first week and then gradually reduced by 2 °C weekly until it reached 22 °C. Relative humidity (RH %) ranged from 65 to 75%. The birds were reared in a naturally ventilated open house with sawdust as litter. Freshwater and feed were offered for ad libitum consumption throughout the experiment. Chicks were housed under the same managerial, hygienic, and environmental conditions all over the experimental period. Usual health and vaccination practices were undertaken against New Castle (at 4th and 14th day) and Gumboro diseases (at 7th and 22 days). The health condition of all the chicks was closely monitored by performing daily health checks. After the study, all remaining birds were released.

### Experimental design and diets

AlphaGal (galactosidase; EC 3.2.1.22) was produced by a genetically modified strain of *Saccharomyces cerevisiae* (Kerry Food Ingredients (Cork) Limited Kilnagleary, Carrigaline, Cork, Ireland). The safety of this enzyme for broiler chicken has been established earlier [37].

Birds were randomly assigned according to a 2 × 2 factorial arrangement consisting of four experimental groups (50 chick /group) with 5 replicates for each treatment (10 chicks /replicate). The experimental group consists of two energy groups: in the first group, the birds were fed on a recommended energy diet (RED) and the birds in the second group were reduced about 120 kcal/kg diet as a low energy diet (LED) and two levels of AlphaGal (0 or 50 mg/kg diet) for RED and LED. The experiment lasted for 35 days and was divided into 3 stages: starter (from 3 to10 days), grower (from 11 to 23 days), and finisher stage (from 24 to 35 days). The formulation and chemical composition of the control groups are shown in (Table 6). The experimental diets were formulated to be iso-energetic iso-nitrogenous by following the standard procedures of Ross 308 broiler nutrition specifications [38].

**Table 6:** The proximate chemical composition of the experimental diets (g/kg)

Ingredients	Unit	Starter		Grower		Finisher	
		RED	LED	RED	LED	RED	LED
Corn 7.25% Cp	g/kg	530.0	513.0	580.0	560.0	640.0	600.0
Soybean Meal 47% Cp	g/kg	370.0	377.0	320.0	320.0	244.0	250.0
Wheat bran 14.5% Cp	g/kg	–	30.0	–	35.0	–	49.0
Corn Gluten Meal 60% Cp	g/kg	35.0	25.0	33.0	28.0	46.0	35.0
Oil (Soya) – E76	g/kg	21.0	20. 0	29.0	19.0	33.0	30.0
Dicalcium Phosphate Dcp 18%	g/kg	20.0	11.0	17.0	17.0	15.5	14.5
Calcium Carbonate – Limestone	g/kg	7.0	7.0	5.0	5.0	6.0	6.0
Dl Methionine 99%	g/kg	3.6	3.7	3.0	3.0	3.2	3.0
Sodium Bicarbonate	g/kg	3.2	3.2	3.0	3.0	3.0	3.0
Broiler Premix*	g/kg	3.0	3.0	3.0	3.0	2.8	3.0
L-LYSINE Hcl 98%	g/kg	3.0	2.9	2.8	2.8	2.4	2.4
Salt	g/kg	2.0	2.0	2.0	2.0	2.2	2.2
L-Threonine 98.5%	g/kg	1.0	1.0	1.0	1.0	0.7	0.7
Choline 60 Veg	g/kg	0.7	0.7	0.7	0.7	0.7	0.7
Phytase enzyme	g/kg	0.5	0.5	0.5	0.5	0.5	0.5
CHEMICAL ANALYSIS							
Moisture	%	11.27	11.25	11.23	11.45	11.17	11.41
Crude protein analysis	%	23.83	23.86	21.68	21.74	19.33	19.37
Lysine	g/kg	14.63	14.77	13.28	13.37	11.52	11.60
Methionine	g/kg	7.25	7.29	6.18	6.15	5.98	6.10
Methionine+cystine	g/kg	10.84	10.88	9.51	9.50	9.04	9.17
Threonine	g/kg	9.95	9.97	9.12	9.13	7.84	7.85
Tryptophan	g/kg	2.78	2.85	2.49	2.54	2.08	2.16
Arginine	g/kg	15.35	15.63	13.77	13.94	11.62	11.95
Valine	g/kg	11.27	11.29	10.25	10.27	9.09	9.11
Calcium	g/kg	10.38	10.44	8.87	8.91	8.67	8.51
Av. Phosphorus poultry	g/kg	5.01	5.16	4.51	4.67	4.17	4.26
Sodium	g/kg	1.83	1.84	1.77	1.77	1.68	1.69
Potassium	g/kg	8.84	9.28	8.05	8.42	6.81	7.41
Cl	g/kg	2.34	2.33	2.34	2.35	2.50	2.48
DEB	meq/kg	237.68	239.83	214.76	213.01	175.12	175.51
Crude fiber	%	2.39	2.64	2.32	2.58	2.18	2.55
ME poultry. (kcal/kg)	Kcal/kg	3002.55	2884.01	3103.69	2983.29	3200.87	3083.78

*Premix per kg of diet: vitamin A, 1500 IU; vitamin D3, 200 IU; vitamin E, 10 mg; vitamin K3, 0.5 mg; thiamine, 1.8 mg; riboflavin, 3.6 mg; pantothenic acid, 10 mg; folicacid, 0.55 mg; pyridoxine, 3.5 mg; niacin, 35 mg; cobalamin, 0.01 mg; biotin, 0.15 mg; Fe, 80 mg; Cu, 8 mg; Mn, 60 mg; Zn, 40 mg; I, 0.35 mg; Se, 0.15 mg

### Growth performance

The average initial body weight was obtained at the 4th day of age and then the body weight was recorded at the end of the starter, grower, and finisher stages (10, 23, and 35 days, respectively) to determine the average body weight of the birds in each group. Bodyweight gain was calculated as the difference between the final body weight during the intended period and the initial weight during the same period. Feed intake of each replicate was recorded as the difference between the feed offered weight and residues left and then divided by the number of birds in each replicate to determine the average feed intake per bird. The feed conversion ratio (FCR) was estimated at the end of each stage [39]. FCR = amount of feed consumed (g)/BWG (g). The relative growth rate (RGR) was calculated at the end of the experiment using the equation [40]. $\mathrm{RGR}=\left[\left(\mathrm{W}2-\mathrm{W}1\right)/\frac{\left(\mathrm{W}1+\mathrm{W}2\right)}{2}\right]\times 100$. The protein efficiency ratio (PER) was determined according to [41] as the number of grams of weight gain per unit of weight of dietary protein consumed. PER = live weight gain (g)/protein intake (g).

### Apparent ileal digestibility of amino acids

#### Diet and birds

For determining the ileal digestibility of amino acids, titanium dioxide, an indigestible marker that does not affect nutrient digestibility and has a high recovery rate of almost 100%, was added to the feed at 0.5% dosage (5 kg/t of feed) for 5 days. Each assay diet was offered ad libitum to five replicates (five birds /replicate) of broiler chicken from 35 to 40 days of age. At the end of the bioassay, all the birds were slaughtered, and the contents of the lower half of the ileum were collected by gently flushing with distilled water into plastic containers. Ileal digesta of the birds within a pen was pooled, frozen immediately after collection, and subsequently freeze-dried. Dried ileal digesta samples were ground to pass through a 0.5 mm sieve and stored in airtight containers at − 20 °C for chemical analyses.

#### Chemical analysis

Amino acid concentrations in the diet and ileal digesta samples were determined using cation exchange column chromatographic procedures with post-column derivatization and fluorimetric detection of amino acids using *0*-phthaldialdehyde as described earlier [42, 43]. Tryptophan was determined separately using the method of Ravindran and Bryden [44]. Titanium dioxide was determined using the methods of Fenton and Fenton [45]. AID coefficients for AA were calculated using the following equation:

$$ \mathrm{AID}\ \left(\%\right)=100-\left[{\left({\mathrm{Ti}}_{\left(\mathrm{diet}\right)}\times {\mathrm{AA}}_{\left(\mathrm{ileum}\right)}\right)}_{/}\ \left({\mathrm{Ti}}_{\left(\mathrm{ileum}\right)}\times {\mathrm{AA}}_{\left(\mathrm{diet}\right)}\right)\times 100\right] $$

Where Ti (diet) is the concentration of titanium dioxide in the diet, Ti (ileal) is the concentration of titanium dioxide in the ileal digesta, AA (ileal) is the concentration of the test amino acid in the ileal digesta, and AA (diet) is the concentration of the test amino acid in the diet.

#### Sample collection and laboratory analyses

At the end of the experiment (at day 35), the birds were made to fast overnight and blood samples were collected from five birds randomly selected from each treatment group (one bird /replicate) after euthanasia by cervical dislocation according to [46]. Blood samples were left to clot at room temperature or in the refrigerator for 1 h and then centrifuged at 3000 rpm for 15 min. The clear supernatant serum was transferred into dry, sterile, and labeled stopper vials and used for biochemical analysis. The samples from the different parts of the gut were collected for histological examination of the gut.

Total cholesterol, triglyceride (TG), high-density lipoprotein-cholesterol, and alkaline phosphatase levels were estimated using colorimetric diagnostic kits of spectrum-bioscience (Egyptian Company for Biotechnology, Cairo, Egypt) by following the methods of Allain et al. [47], McGowan et al. [48], Vassault et al. [49], and Garlich [50] respectively. The Iranian formula of low-density lipoprotein (LDL) = TC/1.19 + TG/1.9–HDL/1.1–38 was used for determining LDL-C levels. Chicken ELISA kits of MyBiosource Co. of CAT.NO. MBS012469, MBS701683, and of ABCAM Co. of CAT. NO. AB157691were used for determining the serum levels of alkaline phosphatase, interleukin 10, and IgM, respectively. Meanwhile, a sandwich enzyme-linked immunosorbent assay (ELISA) kit manufactured by Life Span Biosciences, Inc. of CAT.NO.LS-F9287 was used for determining serum complement 3 level by following manufacturer’s instructions.

#### Histological examination of the small intestine

At the end of the experiment (at day 35), the birds were made to fast overnight and samples from different parts of the small intestine were collected from five birds randomly selected from each treatment group after slaughter. The entire small intestine was isolated and duodenum, jejunum, and ileum were segregated. The organs were washed with saline solution to remove the intestinal contents and fixed immediately in 10% neutral buffered formalin for 24 h. They were then dehydrated in ascending grades of ethanol, cleared in xylene,and embedded in paraffin wax using routine histological techniques. Histological sections (5-μm thick) were stained with hematoxylin-eosin (H&E) to determine the general structure. The periodic acid Schiff (PAS) reagent was used for detecting the presence of neutral mucopolysaccharides while Alcian blue stain was used for detecting the presence of acidic mucopolysaccharides. The methods of processing and staining employed by Bancroft & Gamble were adopted. The stained sections were examined by a standard light microscope and a digital DSc-W 800 super-steady Cybershot camera (Sony, Japan) connected to an Olympus BX21 light microscope.

#### Histomorphometric examination of the small intestine

Quantitative analysis of the height of the villi in duodenum, jejunum, and ileum was performed for each experimental group. Moreover, the depth of the intestinal crypt in different regions was noted. Quantitative analysis was performed using Image J software (Http://rsb.Info.nih.gov/ij/).

#### Economic efficiency

Collective efficiency measures were calculated according to [51, 52]. It includes total return, total costs, variable costs, and net profit. The performance index (PI) was calculated according to an earlier study [53].

### Statistical analysis

The data were analyzed by using SPSS 18.0 for Windows (SPSS Inc., Chicago, IL, USA) and expressed as the mean ± standard error (SE). Variations were assessed by two-way (ANOVA) and factorial analysis was performed on the factors included in the model such as energy level, the presence of AlphaGal, and their association. Post-hoc Tukey’s multiple range tests were performed to compare the differences between the means at 5% probability.

## Data Availability

The datasets used and analysed during the current study available from the corresponding author on reasonable request.

## Abbreviations

AlphaGal: Alpha-galactosidase
AID%: Amino acid ileal digestibility coefficient
RED: Recommended energy diet
LED: Low energy diet
SBM: Soybean meal
BW: Body weight
BWG: Body weight gain
FI: Feed intake
FCR: Feed conversion ratio
RGR: Relative growth rate
PER: Protein efficiency ratio
Ti: Titanium dioxide
AA: Amino acid
PAS: Periodic acid Schiff reagent
LE: Egyptian pound
